# Optimization of the Solvent and In Vivo Administration Route of Auranofin in a Syngeneic Non-Small Cell Lung Cancer and Glioblastoma Mouse Model

**DOI:** 10.3390/pharmaceutics14122761

**Published:** 2022-12-09

**Authors:** Laurie Freire Boullosa, Jinthe Van Loenhout, Christophe Hermans, Ho Wa Lau, Céline Merlin, Elly Marcq, Farnaz Sedigheh Takhsha, Wim Martinet, Guido R. Y. De Meyer, Filip Lardon, Evelien L. J. Smits, Christophe Deben

**Affiliations:** 1 Center for Oncological Research (CORE), Integrated Personalized & Precision Oncology Network (IPPON), University of Antwerp, 2610 Wilrijk, Belgium; 2 Laboratory of Physiopharmacology, Department of Pharmaceutical Sciences, University of Antwerp, 2610 Wilrijk, Belgium

**Keywords:** auranofin, cancer mouse models, glioblastoma, non-small cell lung cancer, drug delivery, oral gavage, solvent, thioredoxin reductase

## Abstract

The antineoplastic activity of the thioredoxin reductase 1 (TrxR) inhibitor, auranofin (AF), has already been investigated in various cancer mouse models as a single drug, or in combination with other molecules. However, there are inconsistencies in the literature on the solvent, dose and administration route of AF treatment in vivo. Therefore, we investigated the solvent and administration route of AF in a syngeneic SB28 glioblastoma (GBM) C57BL/6J and a 344SQ non-small cell lung cancer 129S2/SvPasCrl (129) mouse model. Compared to daily intraperitoneal injections and subcutaneous delivery of AF via osmotic minipumps, oral gavage for 14 days was the most suitable administration route for high doses of AF (10–15 mg/kg) in both mouse models, showing no measurable weight loss or signs of toxicity. A solvent comprising 50% DMSO, 40% PEG300 and 10% ethanol improved the solubility of AF for oral administration in mice. In addition, we confirmed that AF was a potent TrxR inhibitor in SB28 GBM tumors at high doses. Taken together, our results and results in the literature indicate the therapeutic value of AF in several in vivo cancer models, and provide relevant information about AF’s optimal administration route and solvent in two syngeneic cancer mouse models.

## 1. Introduction

Auranofin (AF) is an orally available, lipophilic, organogold compound with a well-known safety profile. Its chemical name is [2,3,4,6-tetra-o-acetyl-L-thio-β-D-glycol-pyranoses-S-(triethyl-phosphine)-gold(I)] in which the triethyl group and tetraacetylthioglucose (TATG) stabilize the gold molecule [1]. Due to its chemistry, AF is considered a relatively stable compound that exists as a monomer in solution and is freely soluble in lipid membranes. Furthermore, it is likely to undergo ligand exchange reactions in the presence of competing thiols [2]. In 1985, AF was approved by the U.S. Food and Drug Administration (FDA) for the treatment of rheumatoid arthritis (RA) under the brand name Ridaura®. To temper RA disease progression, AF is administered as a 3 mg oral capsule once or twice daily, after which 25–30% of the administered gold is detected in the plasma mostly bound to cysteine-34 of albumin [1,3]. Over the past decades, there was a decline in the prescription of AF as an anti-rheumatic drug. Firstly, due to the adverse side effects associated with its long-term use, which were predominantly gastrointestinal problems. Secondly, more effective medication for RA patients became available, and AF was replaced by other disease-modifying anti-rheumatic drugs (DMARDs) or nonsteroidal anti-inflammatory drugs (NSAIDs) [4].

Recently, gold compounds have increasingly been investigated as potential anti-tumor agents based on their medical and chemical properties described in the literature [5]. Scientific studies in the mid-1980s called attention to AF’s antiproliferative effects against cancer cells for the first time [6]. In the following years, AF has received wide attention as a multifunctional compound with anticancer and antibacterial properties, among others [4,7]. Nowadays, AF is mostly under investigation for oncological application through its main mechanism of action, the inhibition of the redox enzyme thioredoxin reductase 1 (TrxR).

To obtain preclinical evidence on AF as a potent anticancer agent, its antineoplastic activity has already been investigated in various in vitro and in vivo models [4,8]. We provide an overview of the in vivo studies that use AF for the treatment of cancer, including the solvent, dose, treatment schedule and administration route that were used (Table 1). However, we noticed that there were inconsistencies on administration parameters of AF in vivo. The in vivo dose of AF varied between 0.1 and 15 mg/kg and were mostly administered via intraperitoneal (i.p.) injections with varying ways of formulation. To a lesser extent, AF has also been administered per os (p.o.) via oral gavage or via vein injections. The most widely used model to investigate the anti-cancer activity of AF in vivo is the tumor xenograft model, whereby human tumor cells are transplanted into immunocompromised nude mice. The majority of studies using this tumor model demonstrated an inhibitory effect of AF on tumor growth. Next to its use as a monotherapy, AF has also been comprehensively tested in combination with other drugs to further enhance its anti-cancer activity in vivo (Table 1). Table 1 highlights the therapeutic value of AF in various in vivo cancer models, but with inconsistencies on several important parameters of in vivo AF administration. Therefore, we studied the optimal solvent to enhance the solubility of AF and three administration routes (i.p. injection, oral gavage and osmotic minipumps) to identify the most optimal method to reduce AF-mediated toxicity and increase its efficacy in a syngeneic SB28 glioblastoma (GBM) C57BL/6J mouse model and 344SQ non-small cell lung cancer (NSCLC) 129S2/SvPasCrl (129) mouse model. Our results provide information about the optimal administration route and in vivo solvent of AF in our syngeneic C57BL/6J and 129-mouse models; oral gavage with a solvent of 50% DMSO, 40% propylene glycol 300 (PEG300) and 10% ethanol were used, respectively. In addition, we wanted to highlight that the in vivo administration route of AF should be carefully considered based on the mouse model and tumor type. Together with the in vivo results in the literature (Table 1), this study highlights the therapeutic value of AF in several in vivo cancer models.

## 2. Materials and Methods

### 2.1. Mice

Male 129S2/SvPasCrl (129) mice, age 6–9 weeks, were obtained from Charles River. Female C57BL/6J mice, age 6–10 weeks were obtained from Jackson Laboratories. All animal care and experimental procedures were approved by the Ethics Committee of the University of Antwerp (2020-32 and 2020-20). Upon arrival, mice were given a 7-day adaptation period before being used in experiments to reduce stress levels. All mice were housed in a temperature-controlled environment with 12 h light/dark cycles and received food and water ad libitum. Mice were checked on a daily basis to inspect health and wellbeing.

### 2.2. Murine Cell Lines

Murine adenocarcinoma lung cancer cell line 344SQ derived from KrasLa1/+p53R172HΔG mice (subcutaneous (s.c.) metastasis) were kindly provided by Jonathan M. Kurie (University of Texas, MD Anderson Cancer Center, Houston, TX, USA). This cell line is syngeneic to the male 129S2/SvPasCrl mice. The 344SQ cells were cultured in RPMI cell culture medium supplemented with 10% FBS and 10 mM L-Glutamine. The murine glioblastoma cell line SB28 (provided by H. Okada, UCSF, San Francisco, CA, USA) is syngeneic to the female C57BL/6J mice. The SB28 cells were cultured in DMEM supplemented with 10% heat-inactivated FBS, 1% HEPES and 1% GlutaMAX. Cell lines were maintained at 37 °C and 5% CO_2_. All cell lines were tested on a routine base for mycoplasma contamination.

### 2.3. Tumor Kinetics and Survival

Prior to injection, 344SQ and SB28 cells were harvested using TrypLE, washed three times with sterile PBS and put through a 70-µm cell strainer to assure single-cell suspension without any contaminants. Next, 129-mice were injected s.c. with 1 × 10^6^ 344SQ cells suspended in 100 µL sterile PBS in the left shaved flank, while C57BL/6J mice were injected at the same position with 1 × 10^6^ SB28 cells in 100 µL sterile PBS. When tumors reached an average size of 40–50 mm^3^, mice were randomized based on tumor size and divided into different treatment groups. Tumor size was measured three times a week using a digital caliper. Tumor volume was calculated using the formula (length × width^2^)/2. Mice were sacrificed when a tumor size of 1500 mm^3^ was reached.

### 2.4. Auranofin Solvent

AF (Sanbio, Uden, Netherlands) was first dissolved in 100% DSMO to obtain a stock concentration of 50 mg/mL. Then, the AF stock was further dissolved in three different solvents composed of either (i) PBS, (ii) 4% (*v/v*) DMSO/10% (*v/v*) ethanol or (iii) 50% (*v/v*) DMSO, 40% (*v/v*) PEG300 and 10% (*v/v*) absolute ethanol to obtain an intermediate stock solution of 2 mg/mL AF for subsequent in vivo administration. A solvent of 4% (*v/v*) DMSO/10% (*v*/*v*) ethanol was used for i.p. injections, while a solvent of 50% (*v/v*) DMSO, 40% (*v/v*) PEG300 and 10% (*v/v*) absolute ethanol was used for AF administration via the osmotic minipumps and oral gavage.

### 2.5. In Vivo Administration of AF

AF was administered via three different routes of administration in SB28 and 344SQ tumor-bearing mice. First, AF was administered via daily i.p. injections over a period of 14 consecutive days. Second, AF was administered s.c. via osmotic minipumps (Alzet, CA, USA, type 1002). In practice, the osmotic minipumps were filled with either 100 µL AF or vehicle (solvent). The required AF concentration for these devices was based on the average weight per treatment group and calculated using the online tool provided by Alzet [47]. Mice were anaesthetized with isoflurane. The hair of the neck was shaved and a small incision was made between the ears. Using a hemostat, a s.c. pocket was made wide enough for an osmotic minipump. The pocket was flushed with saline and an osmotic minipump was inserted. Then, the incision was closed with two sterile surgical staples. The osmotic minipump provided a long-term delivery of 14 days after which it was removed while the mice were under anesthesia. Third, AF was administered daily via oral gavage using a 20G flexible feeding needle for a period of 14 days. Calculations of the required AF concentration in mg/kg were made for each individual mouse based on the individual body weight. The toxicity of AF was measured based on total body weight, behavior, the Mouse Grimace Scale (MGS) and post-mortem evaluation.

### 2.6. Thioredoxin Reductase Activity Assay

After 14 days of AF treatment p.o., SB28 tumors were dissected and disrupted in lysis buffer using a tissue homogenizer (Qiagen, Hilden, Germany). Afterwards, protein lysates were used to measure TrxR activity using the Thioredoxin Reductase Colorimetric Assay kit (Cayman chemical, Ann Arbor, MI, USA) according to the manufacturer’s instructions. Absorbance was recorded at 405 nm with a Spark®Cyto (Tecan, Männedorf, The Switzerland) during the initial 5 min of the reaction. TrxR activity was calculated using the formula provided by the protocol, whereby background measurements were subtracted from all values. An equal amount of protein was loaded for each condition as determined by the Pierce BCA protein kit (Thermo Scientific, Merelbeke, Belgium).

### 2.7. Statistics

Differences were considered to be statistically significant if *p* < 0.05. To analyze differences in tumor kinetics overtime, we used R [48] with afex [49] and emmeans [50] packages to perform mixed model ANOVA. To assess differences between the TrxR activity in the different treatment groups, a Mann–Whitney U test was performed using SPSS v27. Graphs were made using GraphPad v9 software.

## 3. Results

### 3.1. Daily I.P. Injections with AF Induce Weight Loss and Gut-Related Cytotoxicity in 344SQ 129-Mouse Model

**Figure 1:**
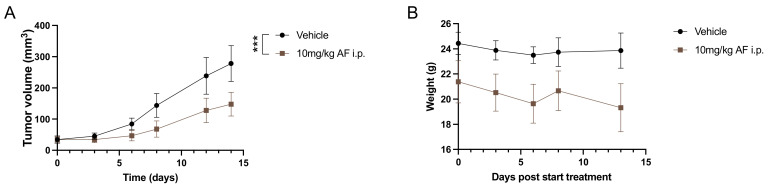
Effect of intraperitoneal (i.p.) injections with a high dose of auranofin (AF) on 344SQ tumor-bearing 129S2/SvPasCrl (129)-mice. (**A**) Tumor growth kinetics of 344SQ tumor-bearing 129-mice after treatment with 10 mg/kg AF via i.p. injections for 14 consecutive days. (**B**) Body weight of 129-mice after daily treatment with 10mg/kg AF via i.p. injections over a period of 14 days. Data represent mean ± SD. N = 5 mice per group. *** *p* ≤ 0.001.

In the literature, i.p. injections with 10 mg/kg AF were most often used for the in vivo administration of AF (Table 1). Therefore, we tested the administration of 10 mg/kg AF via daily i.p. injections over a period of 14 consecutive days in the 344SQ tumor-bearing 129-mouse model. We observed a significant delay in tumor growth in AF-treated mice, as compared to vehicle treated controls (Figure 1A). Moreover, after five days of treatment, mice showed clinical signs of cytotoxicity, discomfort and weight loss of approximately 20% during the treatment period (Figure 1B). The MGS was used to assess post-procedural pain by evaluating changes in the facial expressions of mice [51]. After i.p. injections with AF, mice showed a moderate or marked appearance of 3 out of 5 MGS action units (orbital tightening, ear position and whisker change). Postmortem examination also revealed bloated and obstructed intestines (Figure S1). This cytotoxic effect was not mouse strain-dependent, since C57BL/6J mice (without a tumor) showed the same behavioral changes of the MGS immediately after i.p. injection with 10 mg/kg AF.

In addition, we also encountered some problems with the solubility of AF at doses of 10 mg/kg and above. Dilutions in both PBS and 4% (*v/v*) DMSO/10% (*v/v*) ethanol resulted in precipitations of the compound over time. For future experiments, other routes of AF administration and another solvent should be investigated to improve the solubility of AF.

### 3.2. Continuous Slow Release of AF Treatment in 344SQ- and SB28-Bearing Mice via an Osmotic Minipump

**Figure 2:**
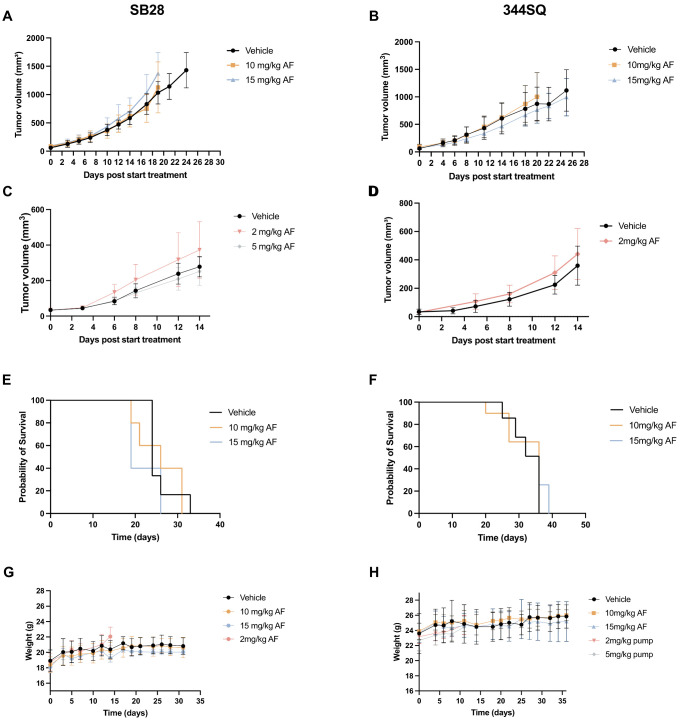
The effect of continuous delivery of low and high doses of AF using a subcutaneous (s.c.) osmotic minipump system on SB28 tumor-bearing C57BL/6J and 344SQ tumor-bearing 129-mice. (**A**,**B**) Tumor growth kinetics over time after 14 days of treatment with high doses of AF (10 or 15 mg/kg) delivered via a s.c. implanted osmotic minipump in SB28 C57BL/6J (**A**) and 344SQ 129 tumor-bearing mice (**B**). (**C**,**D**) Tumor growth kinetics after 14 days of treatment with low doses of AF (2 or 5 mg/kg) delivered via a s.c. implanted osmotic minipump in SB28 C57BL/6J (**C**) and 344SQ 129 tumor-bearing mice (**D**). (**E**,**F**) Survival of SB28 C57BL/6J mice (**E**) and 344SQ 129-mice (**F**) treated with high doses of AF via osmotic minipumps. (**G**,**H**) Body weight of SB28 C57BL/6J mice (**G**) and 344SQ 129-mice (**H**) after treatment with different doses of AF delivered via a s.c. implanted osmotic minipump. Data represent mean ± SD. N = 5 mice per group.

Based on the literature [52] and chemical properties of AF, a mixture of 50% DMSO, 40% PEG300 and 10% absolute ethanol was used to successfully prepare an intermediate AF stock solution of 2 mg/mL without precipitation. To administer AF chronically for 14 days, osmotic minipumps (Alzet, type 1002) were implanted s.c. in both SB28 and 344SQ tumor-bearing mice to deliver 2, 5, 10 and 15 mg/kg AF per day at the same constant rate. Compared to the toxic i.p. injections with AF, these devices are easy to use, provide constant drug plasma levels for 14 days and induced only minimal stress in the animals [52,53]. After 14 days of AF delivery via the minipumps, there was no delay in tumor growth and no increase in survival in both SB28 C57BL/6J and 344SQ 129-mice treated with high doses (10–15 mg/kg) or low doses (2–5 mg/kg) of AF, compared to vehicle-treated mice (Figure 2A–F). The weight of mice in both models remained constant during and after the 14 days of treatment with different doses of AF, and there was no observed toxicity based on behavior or the MGS (Figure 2G–H). However, we observed that s.c. delivery of high doses of AF (10–15 mg/kg) via the osmotic minipumps resulted in skin irritation and lesions at the position of the pump where AF was released (Figure S2). As a result of these lesions, high doses of AF may not have been properly released, whereby no significant positive effect on tumor growth and survival was observed (Figure 2A–F). These skin lesions were not observed when lower doses of AF (2–5 mg/kg) or the vehicle were administered to the mice via the s.c. minipumps.

In conclusion, the mixture of 50% DMSO, 40% PEG300 and 10% absolute ethanol is a good vehicle for dissolving AF at both low and high concentrations. However, neither i.p. injections nor s.c. delivery via osmotic minipumps were the ideal route of administration for AF.

### 3.3. Oral Administration of AF Treatment in 344SQ- and SB28-Bearing Mice

As an anti-rheumatic drug, AF is formulated as a capsule and given orally to patients. To mimic its clinical administration, we changed the administration route of AF to daily oral gavage using a 20G flexible feeding needle for a period of 14 days.

**Figure 3:**
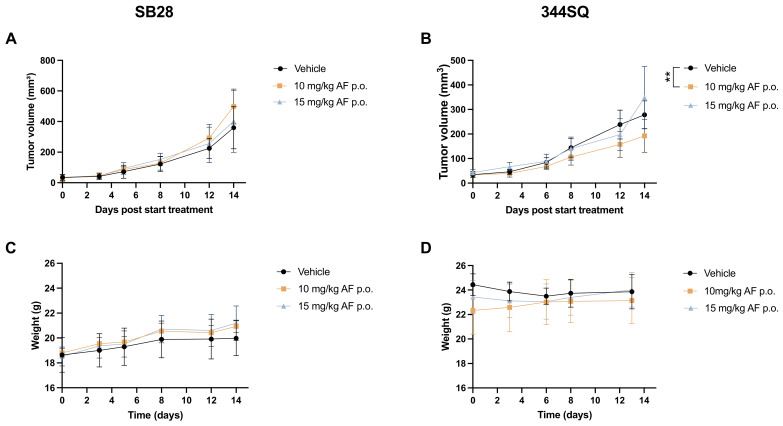
Effect of oral administration of high AF doses on SB28 tumor-bearing C57BL/6J and 344SQ tumor-bearing 129-mice. (**A**,**B**) Tumor growth kinetics of SB28 (**A**) and 344SQ (**B**) tumor-bearing mice after treatment with high AF doses (10–15 mg/kg) via oral gavage for 14 consecutive days. (**C**, **D**) Body weight of SB28 (**C**) and 344SQ (**D**) tumor-bearing mice after daily oral treatment with high AF doses (10–15 mg/kg) for a period of 14 days. Data represent mean ± SD. N = 5 mice per group. ** *p* ≤ 0.01.

After daily treatment with 10 or 15 mg/kg AF for 14 consecutive days via oral gavage, there was no delay in tumor growth in SB28 tumor-bearing C57BL/6J mice compared to the vehicle group (Figure 3A). In the 344SQ 129-mice, results showed a significant decrease in tumor volume after 10 mg/kg AF treatment compared to vehicle (Figure 3B). Nonetheless, there was no observed cytotoxicity based on body weight or behavior in either C57BL/6J or 129-mouse models (Figure 3C,D) and there were no visual signs of local toxicity in the peritoneal cavity or intestines after postmortem dissection.

Compared to i.p. injections and s.c. minipump delivery of AF, oral gavage was the best route of administration for AF in our SB28 tumor-bearing C57BL/6J and 344SQ tumor-bearing 129-mouse models.

### 3.4. AF Inhibits TrxR Activity in SB28 Tumors

**Figure 4:**
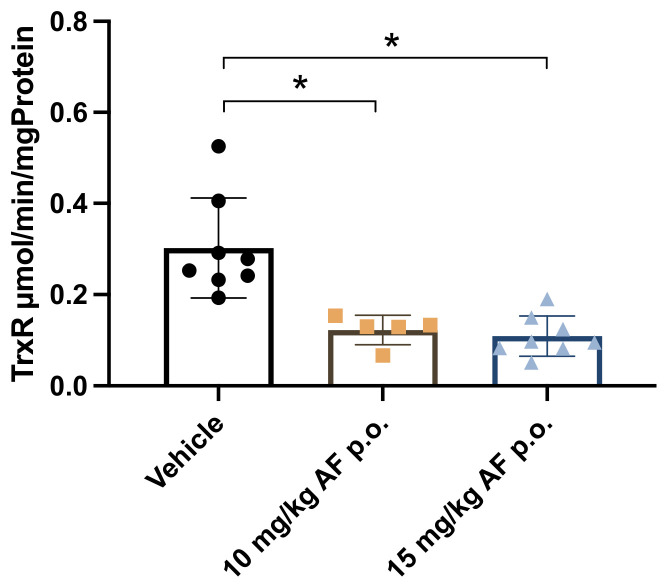
Thioredoxin reductase (TrxR) activity in SB28 tumors of C57BL/6J mice after oral administration of AF. TrxR activity in SB28 tumors isolated from mice after 14 days of daily oral AF treatment. Data represent mean ± SD. N = 5–8 mice per group. * *p* < 0.05 compared to vehicle control.

The main mechanism of action of AF is the inhibition of redox enzyme TrxR. Protein lysates were isolated from disrupted SB28 tumors after 14 days of oral gavage treatment with high AF doses (10 and 15 mg/kg) to check TrxR activity. TrxR activity was significantly decreased in both the 10 mg/kg and 15 mg/kg AF-treated groups compared to the vehicle group (Figure 4).

In conclusion, oral administration of high doses of AF was able to inhibit TrxR activity in SB28 tumors after 14 days.

## 4. Discussion

Due to rising costs, high risk of failure and slow clinical translation of new drug discovery and development, there is an increasing interest in repurposing well-known and well-characterized licensed non-cancer drugs to the oncology domain, as underscored by the Repurposing Drugs in Oncology project [54,55,56]. Hence, the compound AF is receiving increasing interest as an object of repurposing strategies in cancer due to its inhibitory function against TrxR. The therapeutic efficacy of AF against cancer and its relative safety profile in RA patients emphasize the potential of AF as an attractive drug for further clinical investigation.

Aside from multiple in vitro studies, AF is also increasingly tested in various in vivo cancer models to predict its safety, toxicity and efficacy. However, as summarized in Table 1, there is a lack of consistency regarding the dosage, solvent and administration route for AF treatment in mice. Therefore, the goal of this study was to test different delivery methods and solvents for AF treatment in GBM and NSCLC mouse models.

In the literature, the in vivo dose of AF varies between 0.1 and 15 mg/kg and is mostly administered via i.p. injections with different treatment schedules and solvents (Table 1). In a preliminary in vivo experiment with 344SQ tumor-bearing 129-mice, we observed a significant delay in tumor growth after treatment with 10 mg/kg AF via daily i.p. injections for 14 days, compared to vehicle-treated mice. Similarly, several other in vivo studies demonstrated an inhibitory effect of AF monotherapy on tumor growth after i.p. injections in different cancer mouse models [9,10,11,13,15,16,17,18,19,20,21,24,26,30,35,40] (Table 1). In CLL and BCP-ALL xenograft mouse models, i.p. AF injections caused a reduction in the leukemia cell burden and human blasts [9,12]. The reduction in tumor volume was related to AF-mediated induction of apoptotic tumor cell death, as measured by TUNEL assay [22,26] or caspase-3 cleavage [10,21,30], and to a decreased proliferation, as measured by Ki67 staining [11,21,22] in different in vivo models (Table 1). Additionally, a limited number of studies reported that AF treatment via i.p. injections significantly improved the survival of mice compared to mice that received no treatment [9,12]. In studies investigating AF in combination with other clinically applicable compounds in vivo, i.p. injections of AF monotherapy ranging from 0.1 to 10 mg/kg often did not exhibit significant suppression of tumor growth in distinct cancer mouse models [25,28,33,37,38,42,45].

In the present study, we noticed severe overall toxicity in 129-mice after i.p. AF injections, since they lost on average 20% of their body weight. Moreover, 129-mice that were injected i.p. with AF showed visual signs of pain based on the MGS and had bloated and obstructed intestines compared to vehicle-treated mice, as observed in postmortem examination. This effect was not mouse strain dependent, since the C57BL/6J mouse showed the same visual signs of pain and behavior changes after i.p. injections with 10 mg/kg AF. Similarly, the most common adverse effect in about 50% of RA patients treated with AF are gastrointestinal problems such as loose stools, abdominal cramping and watery diarrhea during early months of administration [4,57]. These side effects are controlled by reducing the dosage, or temporary or permanent withdrawal of AF in these patients [57]. However, other studies using i.p. injections for AF delivery in mice did not report on this type of toxicity and even indicate absences of weight loss or blood count anomalies (Table 1). Therefore, we believe it is important that the administration route of AF is carefully considered based on the type of mouse model. Additionally, the prediction of drug cytotoxicity remains a major goal in drug development and the route of drugs to the clinic. It is very important that drugs are nontoxic with minimized side-effects for the patient. However, clinical trials with anticancer drugs often fail due to safety reasons and unmanageable toxicity [56,58]. It is critical at each stage of drug development to consider safety as a primary concern, even if it is not a primary objective [56]. Acute and chronic toxicity of drug candidates, which mimic the clinical dose regimen, are always examined in animal models. The accumulation of drugs in vital organs or blood cells is one the major factors for toxicity [58]. In both our mouse models, we experienced immediate acute toxicity after i.p. injections with AF. Therefore, we choose not to continue with this type of administration in both 129 and C57BL/6 models. We wanted to find an administration route for AF that showed treatment efficacy in vivo without inducing cytotoxic side-effects.

During the formulation of an intermediate stock solution of AF for i.p. injections, we experienced solubility problems when dissolving AF at a concentration of 2 mg/mL, for administration of 10 mg/kg AF or higher to the mice. Both PBS and 4% DMSO/10% ethanol resulted in precipitations of AF over time, even though these solvents were used in other studies (Table 1). In the literature, there is no uniformity on the appropriate solvent for formulation of AF in vivo (Table 1). In the present study, a vehicle composed of 50% DMSO, 40% PEG300 and 10% ethanol was found to improve the solubility of AF and to be nontoxic for mice, based on weight observations and postmortem checks of vital organs. This vehicle has already been shown to be compatible and safe to use for the in vivo delivery of compounds via osmotic minipumps or oral gavage, even with this high ratio of DMSO [52]. In the human setting, the risk of DMSO-mediated toxicity is bypassed since AF, as an anti-rheumatic drug or in clinical trials, is given to patients in oral capsules (3 mg).

Since daily i.p. injections with AF appeared to be toxic in our 344SQ tumor-bearing 129-mouse model, we opted for two different delivery methods. Firstly, we investigated the continuous and slow delivery of low and high doses of AF using osmotic minipumps implanted s.c. in SB28 and 344SQ tumor-bearing mice. We opted for this administration route since these small infusion pumps can provide accurate and continuous dosing of AF to mice [52]. They form a convenient and reliable alternative to the frequent i.p. injections by maximizing therapeutic efficacy and reducing adverse effects. However, high doses of AF (10 and 15 mg/kg) released s.c. via the minipump system induced skin ulceration and rupture at the position of the pump where AF is released. Due to these skin lesions, s.c. administration of AF in the mice was not reliable and no proper absorption at the delivery site was achieved; therefore, AF could not induce the desired effect on tumor growth or survival. AF also induces skin irritations and rash in 20% of RA patients within the first year of treatment [4]. Lower dose concentration of AF did not induce these skin lesions, but was not powerful enough to significantly affect tumor growth. Therefore, we are convinced that higher doses of AF are necessary to induce a strong anticancer effect as a monotherapy in vivo. Secondly, to mimic clinical administration of AF to RA patients, we tested the delivery of AF via oral gavage in our GBM and NSCLC mouse models. High doses of AF p.o. were well-tolerated in both models without any weight loss or visual signs of toxicity. A study by Abutaleb et al. reported that AF was stable after exposure to simulated gastric pH and was not affected by the enzymes of gastric fluids [59]. We showed a significant decrease in tumor volume after 10 mg/kg AF treatment in the 344SQ tumor-bearing 129-mice, but not in SB28 tumor-bearing C57BL/6J mice. In a *TP53*-mutated diffuse large B-cell lymphoma (DLBCL) PDX model, treatment with 50 mg/kg AF via oral gavage for 21 consecutive days also significantly inhibited tumor growth, without any body weight differences [14]. Dependent on the tumor type, AF monotherapy is able to significantly reduce tumor growth in some studies (Table 1). However, targeting different hallmarks of cancer via a combination strategy is a more effective therapeutic approach compared to monotherapies. Therefore, we strongly believe that the true power of AF lies within rationally designed drug combination strategies, as seen by the numerous combination studies in Table 1, to further improve the anti-cancer potential of AF, overcome tumor heterogeneity and limit the ability of cancer cells to adapt and develop treatment resistance. For example, in the same SB28 GBM mouse models, daily oral delivery of AF in combination with another ROS-inducing compound significantly reduced tumor growth and prolonged survival in vivo [45]. In a colorectal xenograft model, daily administration of 6 mg/kg AF via oral gavage in combination with 5-fluorouracil (5-FU) inhibited tumor growth and reduced the number of metastatic lung nodules compared to AF monotherapy [29]. These results confirm the potential of oral administration for AF treatment in vivo in combination regimens.

In vitro experiments already demonstrated that TrxR inhibition is one of the main mechanisms of action of AF [60,61]. Our results confirmed that oral delivery of high doses of AF (10 and 15 mg/kg) led to the inhibition of TrxR activity within SB28 tumors in vivo. This is in-line with other studies showing TrxR inhibition after AF delivery via i.p. injections at varying doses, ranging from 5 mg/kg to 10 mg/kg, using a colorimetric TrxR assay [10,26,33]. Additionally, the combination of AF and the GSH biosynthesis inhibitor BSO also inhibited TrxR activity in vivo, but at lower AF concentrations of around 1.5 mg/kg [31,46]. These data suggest that AF is able to inhibit its primary target TrxR in vivo via both i.p. injections and oral gavage. Despite TrxR inhibition in SB28 tumors, oral administration of AF did not result in the inhibition of SB28 tumor growth. A possible explanation could be that TrxR inhibition was not strong enough and other mechanisms counteracted the perturbed redox status after TrxR inhibition. Our previous in vitro results demonstrated that a low dose of AF-induced TrxR inhibition, subsequently boosted the cell’s antioxidant defense capacities by upregulating pro-survival molecules, such as NRF2 and glutathione, to prevent cancer cell death [62].

The recommended long term dosing regimen of AF in adult RA patients is 6 mg daily, in a single dose or divided doses [1]. The toxicity profile and therapeutic effect of AF was monitored in clinical trials with more than 5000 RA patients taking the drug, plus RA patients that were monitored for over 7 years. Overall, AF did not show any evidence of severe cumulative toxicity in RA patients [59,63]. However, much higher doses of AF, alone or in combination with other drugs, were needed in vivo to inhibit TrxR activity in the tumors of mice, as shown by our results and others (Table 1). Therefore, it might prove challenging to obtain sufficiently high concentrations of AF in the patient’s tumor without increasing unwanted side-effects [4,64,65]. New technological innovations, such as AF-loaded nanoparticles, could offer a possible solution since they are able to enhance drug localization in the target site and minimize systemic cytotoxicity [66,67]. Alternatively, well-designed combination strategies might limit the need for high AF doses. As listed in Table 1, AF in combination with vitamin C, anti-PD-L1, MK2206, adriamycin, cisplatin, buthionine sulfoximine, cold atmospheric plasma etc. showed statistically significant reduction in tumor growth and/or prolonged survival, without obvious side effects, in several in vivo cancer models [25,26,33,35,37,40,41,45].

## 5. Conclusions

Daily i.p. injections of 10 mg/kg AF induced weight loss and gastro-intestinal problems in the 129-mouse model and the use of osmotic pumps resulted in local skin lesions. Therefore, oral gavage was the most appropriate administration route for high doses of AF in the syngeneic GBM C57BL/6J and NSCLC 129-mouse models, without inducing weight loss or showing signs of toxicity. However, it is important to be aware that the administration route of AF should be carefully considered based on the mouse model and type of tumor, since our data show that inappropriate selection can lead to false interpretation of the anti-tumoral effect. A solvent consisting of 50% DMSO, 40% PEG300 and 10% ethanol provided optimal solubility of AF for p.o. administration to mice. In addition, AF was a potent TrxR inhibitor in SB28 GBM tumors at high doses, but failed to inhibit tumor growth. Altogether, our results and in vivo results already described in the literature (Table 1) highlight the therapeutic value of AF in several in vivo cancer models; however, it requires more standardization for its route of administration, and solvent per mouse model and tumor type.

## Figures and Tables

**Table 1:** Overview of the in vivo studies that use Auranofin (AF) for the treatment of cancer.

Treatment	In Vivo Cancer Model	Solvent AF Concentration	Administration Route Treatment Schedule	Experimental Outcome	Ref.
AF MONOTHERAPY					
AF	CLL cells in TCL-1 transgenic mice (Tcl1-tg:p53−/−)	- AF 10 mg/kg	i.p. 5 times a week, for 2 weeks	Reduction in leukemia cell burden and CLL cells in peritoneal cavity Improvement of survival	[9]
AF	RPMI8226 MM xenograft model in NOD/SCID mice	- AF 5 mg/kg	i.p. 5 times a week, for 2 weeks	Inhibition of MM tumor growth Increase in % of apoptotic cells Inhibition of TrxR activity	[10]
AF	KBM5 (Bcr-Abl wild-type) and KBM5-T315I (Bcr-Abl-T315I) CML xenografts in nude mice	10% DMSO, 30% cremophor and 60% NaCl AF 7 mg/kg/day	i.p. 12 days	Inhibition of tumor growth and tumor weight—decrease in proliferative cells (Ki67) Constant body weight	[11]
AF	BCP-ALL xenograft model in NSG mice	DMSO AF 10 mg/kg	i.p. 5 times a week, for 3 weeks	Reduction in number of human blasts Prolongation of event-free survival of leukemic mice	[12]
AF	Hodgkin lymphoma L-540 gemcitabine-resistant-derived tumor xenograft model in nude mice	- AF 10 mg/kg	i.p. 3 times a week, for 17 days	Reduction in tumor growth	[13]
AF	TP53-mutated diffuse large B-cell lymphoma (DLBCL) PDX model	- AF 50 mg/kg	p.o. 21 consecutive days	Reduction in tumor growth No weight loss	[14]
AF	HepG2 liver carcinoma and MCF-7 breast cancer xenograft models in nude mice	10% DMSO, 30% Cremophor EL and 60% NaCl AF 6 mg/kg	i.p. 21 days	Reduction in tumor growth and tumor weight Constant body weight	[15]
AF	CT26 colon cancer xenograft model in BALB/c mice	10% DMSO AF 10 mg/kg	i.p. three times a week for 3 weeks	Reduction in tumor growth Constant body weight	[16]
AF	Calu3 NSCLC tumor xenograft in nude mice	2% DMSO, 10% ethanol and 5% PEG400 AF 10 mg/kg	i.p. -	Significant suppression of tumor growth Constant body weight	[17]
AF	NSCLC PDX model in nude mice	2% DMSO, 10% ethanol, and 5% PEG400 AF 10 mg/kg	i.p. Daily for one week, twice a week for the duration of the experiment	Inhibition of tumor growth Constant body weight	[18]
AF	orthotopic ovarian ES2 TIC tumors in immune-deficient nude mice	dissolved in 100% ethanol and diluted in saline (0.9%) AF 12 mg/kg	i.p. 6 times a week for the duration of the experiment	Significant inhibition of tumor growth Decrease mitotic index	[19]
AF	A2780 orthotopic and s.c. xenograft model in nude mice	- AF 15 mg/kg	i.p. 3 times a week, for 2 weeks	s.c. model: decrease in tumor volume Orthotopic model: no reduction in tumor volume	[20]
AF	22RV1 prostate cancer xenograft model in nude BALB/c mice	10% DMSO, 30% Cremophor EL and 60% normal saline AF 6 mg/kg	i.p. 2 weeks	Reduction in tumor volume and tumor weight—increase cleaved caspase-3 and decrease in proliferation (Ki67) Constant body weight	[21]
AF	R1-DDR prostate cancer xenograft	- AF 5 mg/kg	- 5 times a week, for undefined period	Decrease in tumor volume Higher number of apoptotic cells Decrease in proliferative cells (Ki67) Constant body weight	[22]
COMBINATION THERAPY					
AF + Celecoxib	DLD-1 colon cancer xenograft model in nude mice	Olive oil AF 10 mg/kg	p.o. 30 days (except Saturday and Sunday)	Constant body weight AF mono: moderate therapeutic effect on tumor volume Combo: significant reduction in tumor volume and tumor weight	[23]
AF + sorafenib (+cyclophosphamide)	SK-Hep1OE or SK-Hep1VC liver cancer xenograft model in immune-deficient mice Sorafenib-resistant MV4-11R leukemia xenograft model	- AF 10 mg/kg	i.p. and p.o. 22 days	Reduction tumor growth and tumor weight in both liver and leukemia model Constant body weight (p.o.)	[24]
AF + Vitamin C	MDA-MB-231 breast cancer xenograft model in Crl:NU(Ico)-Foxn1nu nude mice	PBS AF 10 mg/kg	i.p. 5 times a week, for 15 days	AF mono: no effect Combo: reduction in tumor volume Constant body weight	[25]
AF + aPD-L1	MDA-MB-231 breast cancer xenograft model in NOD/SCID mice Murine 4T1.2 syngeneic model in immunocompetent mice TNBC PDX model in NOD/SCID mice	- AF mono: 10 mg/kg Combo: 5 mg/kg AF	i.p. 5 times a week, for 2 weeks	AF mono: reduction in tumor growth and tumor weight—upregulation PD-L1 expression—reduction TrxR activity—increase in apoptotic cells Combo in 4T1.2 model: overcomes resistance to PD-L1	[26]
AF + BSO/radiation	MDA-MB-231 breast cancer xenograft model in nude mice	- AF 1.7 mg/kg	i.p. -	Triple combo: significant reduction in tumor volume compared to vehicle, but not to radiation alone—prolongation of survival 5 % loss of body weight No changes in behavior or activity	[27]
AF + 5Z-7-oxozeaenol	SW620 colon cancer xenograft model in nude mice	Sterile 2.5% DMSO in vegetable oil AF 1.6 mg/kg	i.p. 11 days	AF mono: no effect Combo: no statistically significant effect	[28]
AF + 5-FU	5-FU-resistant SW620 colorectal cancer xenograft model in nude mice	- AF 6 mg/kg	p.o. Daily for 6 weeks	AF Mono: no effect Combo: reduction in tumor growth, tumor weight and metastatic lung nodules Constant body weight	[29]
AF + piperlongumine	SGC-7901 gastric xenograft model in immune-deficient nude mice	- AF 2 mg/kg	i.p. Once per day for 14 days	AF mono: inhibition of tumor growth Combo: more effective inhibition of tumor growth Constant body weight	[30]
AF + BSO + carboplatin	A549 and H292 lung cancer xenograft model in nude mice	Absolute ethanol, cremophor EL in normal saline AF 1.6 mg/kg	i.p. 5 times a week, for 2 weeks	Combo AF + BSO: reduction total GSH and TrxR activity Triple combo: most significant decrease in tumor growth rate Constant body weight No behavioral changes Normal blood analysis	[31]
AF + selenocystine	A549 lung tumor xenograft model in immuno-deficient nude mice	PBS AF 2 mg/kg	Caudal vein injection Every other day for 16 days	Mono AF: no effect on tumor growth or tumor weight—no changes TrxR expression Combo: inhibition of tumor weight and tumor volume—inhibition TrxR expression—higher cleaved caspase activity—inhibition of proliferation (Ki67) Constant body weight	[32]
AF + MK2206	H1993 lung cancer xenograft model in nude mice	- AF 5 mg/kg	i.p. -	Mono AF: no effect on tumor growth—inhibition TrxR activity Combo: significant reduction tumor growth—prolonged survival—inhibition TrxR activity	[33]
AF + erlotinib + TUSC2 gene nanovesicles	wild type EGFR TUSC2-deficient H1299 lung cancer xenograft model in nude mice	PBS AF 10 mg/kg	i.p. 5 times a week, for 2 weeks	Triple combo: inhibits tumor growth and prolongs survival Constant body weight	[34]
AF + adriamycin	A549 lung tumor xenograft in nude mice	PBS AF 10 mg/kg	i.p. 5 times a week, for 6 weeks	AF mono: moderate inhibitory effect on tumor growth Combo: strongest effect on tumor growth and tumor weight Constant body weight	[35]
AF + ibrutinib	H1975 NSCLC tumor xenograft model in nude mice	10% DMSO and 10% PEG400 AF 5 mg/kg	Tail vein injection or i.p. -	AF mono: Inhibition of tumor growth—no effect on survival Combo: significant effect on survival—reduction in tumor growth Constant body weight	[36]
AF + cisplatin	H69 SCLC xenograft model in nude mice	DMSO AF 10 mg/kg	i.p. Every two days for 4 weeks	Mono AF: no effect on tumor growth or tumor weight Combo: significant effect on tumor growth and tumor weight—decrease proliferation marker Ki67 Constant body weight	[37]
AF + disulfiram	HepG2 or SMMC-7721 hepatocarcinoma xenograft models in nude mice	10% DMSO, 30% Cremophor EL and 60% normal saline AF 3 mg/kg	i.p. 15 days	AF mono: no effect on tumor size or tumor weight Combo: significant effect on tumor size and tumor —increase in cleaved caspase-3 Constant body weight	[38]
AF + sorafenib	Tail-vein injection model of CRISPR-Cas9-KO p53 and Pten plasmids in immune-competent C57BL/6N mice Orthotopic tumor model of luciferase-labeled HCC MHCC97L cells implanted in nude mice	5% PEG400 + 5% Tween-80 in H_2_O AF 6 mg/kg AF 3 mg/kg	i.p. 14 days 35 days	AF mono: suppression of tumor growth Combo: strongest inhibition of HCC tumor formation and metastases to the lungs Constant body weight	[39]
AF + BSO	Cal-27 HNC xenograft model in nude mice	Saline AF 1 mg/kg	i.p. Every day, for 10 days	AF mono: significant reduction in tumor volume Combo: strongest significant reduction in tumor volume Constant body weight	[40]
AF + BSO + trigonelline	HN3-cisplatin resistant HNC xenograft model in nude mice	- AF 2 mg/kg	i.p. -	AF mono: significant reduction in tumor volume Triple combination: strongest significant reduction in tumor volume and tumor weight—strong increase in apoptotic cells Constant body weight	[41]
AF + vorinostat/rapamycin	KHOS/NP osteosarcoma xenograft model in nude-Foxn1nu mice (canine) Abrams osteosarcoma xenograft model in nude-Foxn1nu mice	DMSO AF 0.1 mg/kg	i.p. 5 times a week, for 3 weeks	AF mono in both models: no effect on tumor size Combo in both models: significant suppression of tumor growth—decrease in proliferative cells (Ki67—increase in apoptotic cells (cleaved caspase-3)	[42]
AF + ganetespib	A673 Ewing sarcoma xenograft model in nude mice (injected intramuscularly proximal to tibia)	0.5% Hydroxypropyl methylcellulose (HPMC, viscosity grade K4M) in 5% dextrose in water AF 12 mg/kg	i.p. 5 times a week	Combo: significant difference in survival rates compared to AF mono and vehicle Constant body weight—no side effects	[43]
AF + 2DG + BSO + radiation	SiHa or CaSki cervical cancer xenograft model in nude mice	0.05% DMSO AF 1.5 mg/kg	i.p. 3 times per week, for period of 35 days	AF + BSO/AF + BSO + 2DG: significant reduction in tumor volume Triple combo + radiation: strong radio-sensitization Constant body weight—no behavioral changes	[44]
AF + plasma	s.c. injection of murine SB28 GBM cell lines in syngeneic C57BL/6J mice model	50% DMSO, 40% PEG300 and 10% ethanol AF 15 mg/kg	p.o. 2 weeks	AF mono: no effect on tumor volume Combo: significant decrease in tumor volume	[45]
AF + BSO + 2DG	Vari068 TNBC xenograft model (injected in mammary fat pads) in NOD/SCID mice Luciferase-labeled SUM159 breast cancer xenograft model in NOD/SCID mice (cardiac injection for metastasis formation)	- AF 1.5 mg/kg	i.p. every 2 days, for 7 weeks	AF + BSO/AF + BSO + 2DG: significant reduction in tumor growth—reduction in TrxR activity and rat—of GSH/GSSG—significant inhibition of metastasis formation	[46]

Abbreviations: CLL, chronic lymphocytic leukemia; MM, multiple myeloma; CML, chronic myelogenous leukemia; NSG, NOD scid gamma mouse; BCP-ALL, B cell precursor acute lymphoblastic leukemia; NSCLC, non-small cell lung cancer; PEG, polyethylene glycol; SCLC, small cell lung cancer; s.c., subcutaneous; HCC, hepatocellular carcinoma; HNC, head and neck cancer; GBM, glioblastoma; PDX, patient-derived tumor xenograft; 2DG, 2-Deoxy glucose; TNBC, triple negative breast cancer; BSO, buthionine sulfoximine.

## Data Availability

Not applicable.

## Supplementary Materials

The following supporting information can be downloaded at: https://www.mdpi.com/article/10.3390/pharmaceutics14122761/s1, Figure S1: Effect of daily i.p. injections with 10 mg/kg AF on the intestinal tract of 129-mice.; Figure S2: Effect of continuous delivery of 10 mg/kg AF using s.c. osmotic minipump system on the skin of C57BL/6J mice.

## References

[1] Kean W.F., Hart L., Buchanan W.W. (1997). Auranofin. Br. J. Rheumatol..

[2] Crooke S.T., Snyder R.M., Butt T.R., Ecker D.J., Allaudeen H.S., Monia B., Mirabelli C.K. (1986). Cellular and molecular pharmacology of auranofin and related gold complexes. Biochem. Pharmacol..

[3] Christodoulou J., Sadler P.J., Tucker A. (1994). A new structural transition of serum albumin dependent on the state of Cys34. Detection by 1H-NMR spectroscopy. Eur. J. Biochem..

[4] Roder C., Thomson M.J. (2015). Auranofin: Repurposing an old drug for a golden new age. Drugs RD.

[5] Tiekink E. (2003). Gold compounds in medicine: Potential anti-tumour agents. Gold Bull..

[6] Mirabelli C.K., Johnson R.K., Sung C.M., Faucette L., Muirhead K., Crooke S.T. (1985). Evaluation of the in vivo antitumor activity and in vitro cytotoxic properties of auranofin, a coordinated gold compound, in murine tumor models. Cancer Res..

[7] Madeira J.M., Gibson D.L., Kean W.F., Klegeris A. (2012). The biological activity of auranofin: Implications for novel treatment of diseases. Inflammopharmacology.

[8] Gamberi T., Chiappetta G., Fiaschi T., Modesti A., Sorbi F., Magherini F. (2022). Upgrade of an old drug: Auranofin in innovative cancer therapies to overcome drug resistance and to increase drug effectiveness. Med. Res. Rev..

[9] Fiskus W., Saba N., Shen M., Ghias M., Liu J., Gupta S.D., Chauhan L., Rao R., Gunewardena S., Schorno K. (2014). Auranofin induces lethal oxidative and endoplasmic reticulum stress and exerts potent preclinical activity against chronic lymphocytic leukemia. Cancer Res..

[10] Sze J.H., Raninga P.V., Nakamura K., Casey M., Khanna K.K., Berners-Price S.J., Di Trapani G., Tonissen K.F. (2020). Anticancer activity of a Gold(I) phosphine thioredoxin reductase inhibitor in multiple myeloma. Redox Biol..

[11] Chen X., Shi X., Zhao C., Li X., Lan X., Liu S., Huang H., Liu N., Liao S., Zang D. (2014). Anti-rheumatic agent auranofin induced apoptosis in chronic myeloid leukemia cells resistant to imatinib through both Bcr/Abl-dependent and -independent mechanisms. Oncotarget.

[12] Fidyt K., Pastorczak A., Goral A., Szczygiel K., Fendler W., Muchowicz A., Bartlomiejczyk M.A., Madzio J., Cyran J., Graczyk-Jarzynka A. (2019). Targeting the thioredoxin system as a novel strategy against B-cell acute lymphoblastic leukemia. Mol. Oncol..

[13] Celegato M., Borghese C., Casagrande N., Mongiat M., Kahle X.U., Paulitti A., Spina M., Colombatti A., Aldinucci D. (2015). Preclinical activity of the repurposed drug auranofin in classical Hodgkin lymphoma. Blood.

[14] Wang J., Wang J., Lopez E., Guo H., Zhang H., Liu Y., Chen Z., Huang S., Zhou S., Leeming A. (2019). Repurposing auranofin to treat TP53-mutated or PTEN-deleted refractory B-cell lymphoma. Blood Cancer J..

[15] Liu N., Li X., Huang H., Zhao C., Liao S., Yang C., Liu S., Song W., Lu X., Lan X. (2014). Clinically used antirheumatic agent auranofin is a proteasomal deubiquitinase inhibitor and inhibits tumor growth. Oncotarget.

[16] Nag D., Bhanja P., Riha R., Sanchez-Guerrero G., Kimler B.F., Tsue T.T., Lominska C., Saha S. (2019). Auranofin Protects Intestine against Radiation Injury by Modulating p53/p21 Pathway and Radiosensitizes Human Colon Tumor. Clin. Cancer Res..

[17] Li H., Hu J., Wu S., Wang L., Cao X., Zhang X., Dai B., Cao M., Shao R., Zhang R. (2016). Auranofin-mediated inhibition of PI3K/AKT/mTOR axis and anticancer activity in non-small cell lung cancer cells. Oncotarget.

[18] Yan X., Zhang X., Wang L., Zhang R., Pu X., Wu S., Li L., Tong P., Wang J., Meng Q.H. (2019). Inhibition of Thioredoxin/Thioredoxin Reductase Induces Synthetic Lethality in Lung Cancers with Compromised Glutathione Homeostasis. Cancer Res..

[19] Wang Y., Hill K.S., Fields A.P. (2013). PKCι maintains a tumor-initiating cell phenotype that is required for ovarian tumorigenesis. Mol. Cancer Res..

[20] Marzo T., Massai L., Pratesi A., Stefanini M., Cirri D., Magherini F., Becatti M., Landini I., Nobili S., Mini E. (2019). Replacement of the Thiosugar of Auranofin with Iodide Enhances the Anticancer Potency in a Mouse Model of Ovarian Cancer. ACS Med. Chem. Lett..

[21] Liu N., Guo Z., Xia X., Liao Y., Zhang F., Huang C., Liu Y., Deng X., Jiang L., Wang X. (2019). Auranofin lethality to prostate cancer includes inhibition of proteasomal deubiquitinases and disrupted androgen receptor signaling. Eur. J. Pharmacol..

[22] Xu J., Yang X., Deshmukh D., Chen H., Fang S., Qiu Y. (2020). The Role of Crosstalk between AR3 and E2F1 in Drug Resistance in Prostate Cancer Cells. Cells.

[23] Han Y., Chen P., Zhang Y., Lu W., Ding W., Luo Y., Wen S., Xu R., Liu P., Huang P. (2019). Synergy between Auranofin and Celecoxib against Colon Cancer In Vitro and In Vivo through a Novel Redox-Mediated Mechanism. Cancers.

[24] Liu X., Zhang Y., Lu W., Han Y., Yang J., Jiang W., You X., Luo Y., Wen S., Hu Y. (2020). Mitochondrial TXNRD3 confers drug resistance via redox-mediated mechanism and is a potential therapeutic target in vivo. Redox Biol..

[25] Hatem E., Azzi S., El Banna N., He T., Heneman-Masurel A., Vernis L., Baïlle D., Masson V., Dingli F., Loew D. (2018). Auranofin/Vitamin C: A Novel Drug Combination Targeting Triple-Negative Breast Cancer. J. Natl. Cancer. Inst..

[26] Raninga P.V., Lee A.C., Sinha D., Shih Y.-Y., Mittal D., Makhale A., Bain A.L., Nanayakarra D., Tonissen K.F., Kalimutho M. (2020). Therapeutic cooperation between auranofin, a thioredoxin reductase inhibitor and anti-PD-L1 antibody for treatment of triple-negative breast cancer. Int. J. Cancer.

[27] Rodman S.N., Spence J.M., Ronnfeldt T.J., Zhu Y., Solst S.R., O’Neill R.A., Allen B.G., Guan X., Spitz D.R., Fath M.A. (2016). Enhancement of Radiation Response in Breast Cancer Stem Cells by Inhibition of Thioredoxin- and Glutathione-Dependent Metabolism. Radiat. Res..

[28] Hrabe J.E., O’Leary B.R., Fath M.A., Rodman S.N., Button A.M., Domann F.E., Spitz D.R., Mezhir J.J. (2015). Disruption of thioredoxin metabolism enhances the toxicity of transforming growth factor β-activated kinase 1 (TAK1) inhibition in KRAS-mutated colon cancer cells. Redox Biol..

[29] Liu C., Zhao Y., Wang J., Yang Y., Zhang Y., Qu X., Peng S., Yao Z., Zhao S., He B. (2020). FoxO3 reverses 5-fluorouracil resistance in human colorectal cancer cells by inhibiting the Nrf2/TR1 signaling pathway. Cancer Lett..

[30] Zou P., Chen M., Ji J., Chen W., Chen X., Ying S., Zhang J., Zhang Z., Liu Z., Yang S. (2015). Auranofin induces apoptosis by ROS-mediated ER stress and mitochondrial dysfunction and displayed synergistic lethality with piperlongumine in gastric cancer. Oncotarget.

[31] Fath M.A., Ahmad I.M., Smith C.J., Spence J., Spitz D.R. (2011). Enhancement of Carboplatin-Mediated Lung Cancer Cell Killing by Simultaneous Disruption of Glutathione and Thioredoxin Metabolism. Clin. Cancer Res..

[32] Fan C., Zheng W., Fu X., Li X., Wong Y.S., Chen T. (2014). Enhancement of auranofin-induced lung cancer cell apoptosis by selenocystine, a natural inhibitor of TrxR1 in vitro and in vivo. Cell Death Dis..

[33] Dai B., Yoo S.-Y., Bartholomeusz G., Graham R.A., Majidi M., Yan S., Meng J., Ji L., Coombes K., Minna J.D. (2013). KEAP1-dependent synthetic lethality induced by AKT and TXNRD1 inhibitors in lung cancer. Cancer Res..

[34] Xiaobo C., Majidi M., Feng M., Shao R., Wang J., Zhao Y., Baladandayuthapani V., Song J., Fang B., Ji L. (2016). TUSC2(FUS1)-erlotinib Induced Vulnerabilities in Epidermal Growth Factor Receptor(EGFR) Wildtype Non-small Cell Lung Cancer(NSCLC) Targeted by the Repurposed Drug Auranofin. Sci. Rep..

[35] Hou G.-X., Liu P.-P., Zhang S., Yang M., Liao J., Yang J., Hu Y., Jiang W.-Q., Wen S., Huang P. (2018). Elimination of stem-like cancer cell side-population by auranofin through modulation of ROS and glycolysis. Cell Death Dis..

[36] Hu J., Zhang H., Cao M., Wang L., Wu S., Fang B. (2018). Auranofin Enhances Ibrutinib’s Anticancer Activity in EGFR-Mutant Lung Adenocarcinoma. Mol. Cancer Ther..

[37] Liu X., Wang W., Yin Y., Li M., Li H., Xiang H., Xu A., Mei X., Hong B., Lin W. (2019). A high-throughput drug screen identifies auranofin as a potential sensitizer of cisplatin in small cell lung cancer. Investig. New Drugs.

[38] Huang H., Liao Y., Liu N., Hua X., Cai J., Yang C., Long H., Zhao C., Chen X., Lan X. (2016). Two clinical drugs deubiquitinase inhibitor auranofin and aldehyde dehydrogenase inhibitor disulfiram trigger synergistic anti-tumor effects in vitro and in vivo. Oncotarget.

[39] Lee D., Xu I.M.-J., Chiu D.K.-C., Leibold J., Tse A.P.-W., Bao M.H.-R., Yuen V.W.-H., Chan C.Y.-K., Lai R.K.-H., Chin D.W.-C. (2019). Induction of Oxidative Stress Through Inhibition of Thioredoxin Reductase 1 Is an Effective Therapeutic Approach for Hepatocellular Carcinoma. Hepatology.

[40] Sobhakumari A., Love-Homan L., Fletcher E.V., Martin S.M., Parsons A.D., Spitz D.R., Knudson C.M., Simons A.L. (2012). Susceptibility of human head and neck cancer cells to combined inhibition of glutathione and thioredoxin metabolism. PLoS ONE.

[41] Roh J.-L., Jang H., Kim E.H., Shin D. (2016). Targeting of the Glutathione, Thioredoxin, and Nrf2 Antioxidant Systems in Head and Neck Cancer. Antioxid. Redox Signal..

[42] Parrales A., McDonald P., Ottomeyer M., Roy A., Shoenen F.J., Broward M., Bruns T., Thamm D.H., Weir S.J., Neville K.A. (2018). Comparative oncology approach to drug repurposing in osteosarcoma. PLoS ONE.

[43] Pessetto Z.Y., Chen B., Alturkmani H., Hyter S., Flynn C.A., Baltezor M., Ma Y., Rosenthal H.G., Neville K.A., Weir S.J. (2017). In silico and in vitro drug screening identifies new therapeutic approaches for Ewing sarcoma. Oncotarget.

[44] Rashmi R., Huang X., Floberg J.M., Elhammali A.E., McCormick M.L., Patti G.J., Spitz D.R., Schwarz J.K. (2018). Radioresistant Cervical Cancers Are Sensitive to Inhibition of Glycolysis and Redox Metabolism. Cancer Res..

[45] Van Loenhout J., Freire Boullosa L., Quatannens D., De Waele J., Merlin C., Lambrechts H., Lau H.W., Hermans C., Lin A., Lardon F. (2021). Auranofin and Cold Atmospheric Plasma Synergize to Trigger Distinct Cell Death Mechanisms and Immunogenic Responses in Glioblastoma. Cells.

[46] Luo M., Shang L., Brooks M.D., Jiagge E., Zhu Y., Buschhaus J.M., Conley S., Fath M.A., Davis A., Gheordunescu E. (2018). Targeting Breast Cancer Stem Cell State Equilibrium through Modulation of Redox Signaling. Cell Metab..

[47] Alzet. https://www.alzet.com/formulating-the-solution/.

[48] Team R.C. (2018). R: A Language and Environment for Statistical Computing.

[49] Singmann H., Bolker B., Westfall J., Aust F. (2019). Afex: Analysis of Factorial Experiments, R Package Version 0.25-1.

[50] Lenth R. (2019). Emmeans: Estimated Marginal Means, aka Least-Squares Means, R Package Version 1.4.3.01.

[51] Langford D.J., Bailey A.L., Chanda M.L., Clarke S.E., Drummond T.E., Echols S., Glick S., Ingrao J., Klassen-Ross T., Lacroix-Fralish M.L. (2010). Coding of facial expressions of pain in the laboratory mouse. Nat. Methods.

[52] Kurdi A., De Doncker M., Leloup A., Neels H., Timmermans J.-P., Lemmens K., Apers S., De Meyer G.R.Y., Martinet W. (2016). Continuous administration of the mTORC1 inhibitor everolimus induces tolerance and decreases autophagy in mice. Br. J. Pharmacol..

[53] Cameron A.M., Adams D.H., Greenwood J.E., Anderson P.J., Cowin A.J. (2014). A Novel Murine Model of Hypertrophic Scarring Using Subcutaneous Infusion of Bleomycin. Plast. Reconstr. Surg..

[54] Pantziarka P., Verbaanderd C., Sukhatme V., Rica Capistrano I., Crispino S., Gyawali B., Rooman I., Van Nuffel A.M., Meheus L., Sukhatme V.P. (2018). ReDO_DB: The repurposing drugs in oncology database. Ecancermedicalscience.

[55] Shim J.S., Liu J.O. (2014). Recent advances in drug repositioning for the discovery of new anticancer drugs. Int. J. Biol. Sci..

[56] Fogel D.B. (2018). Factors associated with clinical trials that fail and opportunities for improving the likelihood of success: A review. Contemp. Clin. Trials Commun..

[57] Chaffman M., Brogden R.N., Heel R.C., Speight T.M., Avery G.S. (1984). Auranofin. A preliminary review of its pharmacological properties and therapeutic use in rheumatoid arthritis. Drugs.

[58] Sun D., Gao W., Hu H., Zhou S. (2022). Why 90% of clinical drug development fails and how to improve it?. Acta Pharm. Sin. B.

[59] Abutaleb N.S., Seleem M.N. (2020). Auranofin, at clinically achievable dose, protects mice and prevents recurrence from Clostridioides difficile infection. Sci. Rep..

[60] Gandin V., Fernandes A.P., Rigobello M.P., Dani B., Sorrentino F., Tisato F., Björnstedt M., Bindoli A., Sturaro A., Rella R. (2010). Cancer cell death induced by phosphine gold(I) compounds targeting thioredoxin reductase. Biochem. Pharmacol..

[61] Zhang X., Selvaraju K., Saei A.A., D’Arcy P., Zubarev R.A., Arnér E.S.J., Linder S. (2019). Repurposing of auranofin: Thioredoxin reductase remains a primary target of the drug. Biochimie.

[62] Freire Boullosa L., Van Loenhout J., Flieswasser T., De Waele J., Hermans C., Lambrechts H., Cuypers B., Laukens K., Bartholomeus E., Siozopoulou V. (2021). Auranofin reveals therapeutic anticancer potential by triggering distinct molecular cell death mechanisms and innate immunity in mutant p53 non-small cell lung cancer. Redox Biol..

[63] Blodgett R.C., Pietrusko R.G. (1986). Long-term efficacy and safety of auranofin: A review of clinical experience. Scand. J. Rheumatol. Suppl..

[64] Laxer R.M., Cassidy J.T., Petty R.E., Laxer R.M., Lindsley C.B. (2005). CHAPTER 5—PHARMACOLOGY AND DRUG THERAPY. Textbook of Pediatric Rheumatology.

[65] Aronson J.K. (2016). Gold and Gold Salts. Meyler’s Side Effects of Drugs.

[66] Díez-Martínez R., García-Fernández E., Manzano M., Martínez Á., Domenech M., Vallet-Regí M., García P. (2016). Auranofin-loaded nanoparticles as a new therapeutic tool to fight streptococcal infections. Sci. Rep..

[67] Awasthi R., Roseblade A., Hansbro P.M., Rathbone M.J., Dua K., Bebawy M. (2018). Nanoparticles in Cancer Treatment: Opportunities and Obstacles. Curr. Drug Targets.

