# Supplementary material for: Optimization of the Solvent and In Vivo Administration Route of Auranofin in a Syngeneic Non-Small Cell Lung Cancer and Glioblastoma Mouse Model
Source: Pharmaceutics. 2022 Dec 9;14(12):2761. doi: 10.3390/pharmaceutics14122761 (PMC9783082; doi:10.3390/pharmaceutics14122761)
Supplement: Supplementary file 1 [file pharmaceutics-14-02761-s001.zip › pharmaceutics-2019619-supplementary.docx]

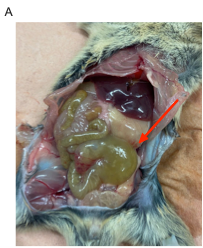


**Figure S1. Effect of daily i.p. injections with 10 mg/kg AF on the intestinal tract of 129-mice.** (**A**) Representative image of postmortem examination of a 129-mouse after daily treatment with 10 mg/kg AF via i.p. injections for 14 days. Red arrow indicates bloated intestines.

**Figure S2. Effect of continuous delivery of 10 mg/kg AF using s.c. osmotic minipump system on the skin of C57BL/6J mice.** (**A**) Representative image of skin lesions in C57BL/6J mice caused by a high dose of 10 mg/kg AF delivered via s.c. minipumps for 14 days.
